# Application of MWD Sensor System in Auger for Real-Time Monitoring of Soil Resistance During Pile Drilling

**DOI:** 10.3390/s25165095

**Published:** 2025-08-16

**Authors:** Krzysztof Trojnar, Aleksander Siry

**Affiliations:** 1 Road and Bridges Department, The Faculty of Civil and Environmental Engineering and Architecture, Rzeszów University of Technology, 12 Powstańców Warszawy Ave., 35-959 Rzeszów, Poland; 2 Independend Researcher, 20 Przemysłowa St., 39-200 Dębica, Poland; aleksandersiry@gmail.com

**Keywords:** measurement while drilling (MWD), Electro-Geo-Probe (EGP), drilled displacement piles (DDP), Cone Penetration Test (CPT), quality control/assessment (QC/QA)

## Abstract

Measuring-while-drilling (MWD) techniques have great potential for use in geotechnical engineering research. This study first addresses the current use of MWD, which consists of recording data using sensors in a drilling machine operating on site. It then addresses the currently unsolved problems of quality control in drilled piles and assessments of their interaction with the soil under load. Next, an original method of drilling displacement piles using a special EGP auger (Electro-Geo-Probe) is presented. The innovation of this new drilling system lies in the placement of the sensors inside the EGP auger in the soil. These innovative sensors form an integrated measurement system, enabling improved real-time control during pile drilling. The most original idea is the use of a Cone Penetration Test (CPT) probe that can be periodically and remotely inserted at a specific depth below the pile base being drilled. This new MWD-EGP system with cutting-edge sensors to monitor the soil’s impact on piles during drilling revolutionizes pile drilling quality control. Furthermore, implementing this in-auger sensor system is a step towards the development of digital drilling rigs, which will provide better pile quality thanks to solutions based on the results of real-time, on-site soil testing. Finally, examples of measurements taken with the new sensor-equipped auger and a preliminary interpretation of the results in non-cohesive soils are presented. The obtained data confirm the usefulness of the new drilling system for improving the quality of piles and advancing research in geotechnical engineering.

## 1. Introduction

Modern MWD (measuring-while-drilling) techniques allow for better assessment of soil properties and more accurate recognition of phenomena caused by pile installation under specific soil conditions and at a given depth. The operation of MWD systems is based on the simultaneous recording and visualization of large amounts of drilling data obtained using sensors [1,2,3]. In the drill rig cabin, computer systems with monitors help the operator control the drilling equipment on the construction site [4,5]. Automatic machine support reduces the failure rates and minimizes wear and tear on specialized tools, e.g., hi-tech augers and innovative drill bits [6,7,8]. As standard, various on-board monitoring systems are installed in pile drilling rigs, which can be used to optimize pile works [9,10,11]. Due to the rapid development of measurement technology and new sensors, MWD can be considered an ideal research method, with the development of new MWD techniques being very useful in geotechnical engineering [1,12]. Research by many authors shows that pile–soil interactions, especially at the pile side and base, are difficult to evaluate technically, analytically, and statistically [13,14,15]. Some analyses have shown that, when determining the bearing capacity of a pile, the method by which the CPT results are averaged is of great importance [16,17]. A problem lies in the difficulty of predicting the soil layers’ positions and the aforementioned challenges in determining the interaction between the pile and soil, as well as the difficulty in determining changes in non-cohesive soil characteristics (favorable or unfavorable) caused by the technological effects of drilling with different augers and different methods of concreting the pile in the ground [18,19].

A new method of drilling displacement piles (DDPs) using special sensors in an EGP auger, as described in this paper, indicates the future direction of MWD technique development. Applying this approach to ground measurement allows for the significant advancement of our knowledge of pile–soil interactions and significantly improves quality control for almost every pile on any construction site. The aim of this study is to discuss our experience with full-scale applications of the innovative EGP auger with an internal sensor system that can support the development of digital drilling rigs guided by artificial intelligence.

## 2. Materials and Methods

### 2.1. MWD Development

Measurement while drilling (MWD) is a research technique that involves continuously recording the drilling process in the ground and automatically monitoring the quality of the drilling work, including drilled piles. The usefulness of MWD lies primarily in recording and archiving large amounts of data obtained using sensors in various parts of a drilling rig. The primary purpose of these measurements is to quantitatively assess the soil resistance during drilling and identify the drilled soil layers. MWD can also be used to gain a better understanding of the effects of pile installation in the ground, optimize the pile production process, and minimize the risks when drilling in weak soils.

The main advantages of MWD are as follows:

- Sensors installed in the drilling rig do not interfere with the pile drilling process.
- Continuous measurement enables real-time control of the geotechnical profile and identification of specific drilling problems in weak soils.
- Automatic collection of large drilling data sets allows for their easy transfer and use in various emerging engineering fields, such as artificial intelligence, CAD, BIM, the IoT, Big Data, Digital Twins, and Interactive Visualizations.

**Figure 1:**
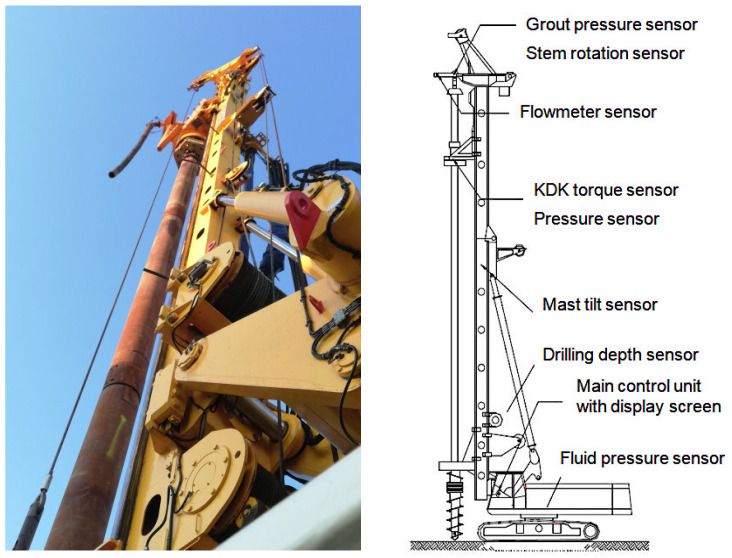
The standard MWD sensor system in the drilling rig.

MWD data can be divided into three main categories depending on the specifics of the data source: mandatory data (e.g., type of drilling rig, drilling method), subjective data (e.g., rig operator’s actions), and objective data (e.g., resistance of the drilled ground, geotechnical layer layout). MWD data can be qualitative or quantitative, but its full usefulness is limited to certain types of soil and piling technologies. Such data is very useful for quality control and assurance (QC/QA) of pile installation in the ground and improving the drilling efficiency under specific soil conditions. The most commonly used data obtained during pile drilling operations includes the drilling depth, penetration, torque and rotation speed, KDK head pressure, downward drilling speed, and upward lift drill speed (during concreting). A typical MWD system, a diagram of which is shown in Figure 1, consists of sensors located in various parts of the drilling rig.

### 2.2. Challenges in Pile–Soil Interaction Control Using MWD

The widespread adoption of MWD techniques in pile drilling is still in its early stages. In some countries, initial standard recommendations and technical requirements for the use of MWD data are already being introduced [20,21,22]. Research and analyses are also being conducted to identify the coefficients of correlations between the various parameters recorded during drilling. Some parameters, such as the rotation speed, penetration rate, thrust, and torque, can be converted into so-called normalized substitute parameters [2,23]. This makes it easier to assess the quality of drilling in a given soil. However, these parameters are more closely related to the operating characteristics of the drilling machine under specific soil conditions, and therefore they are less reliable for evaluating the quality of pile foundation work.

**Figure 2:**
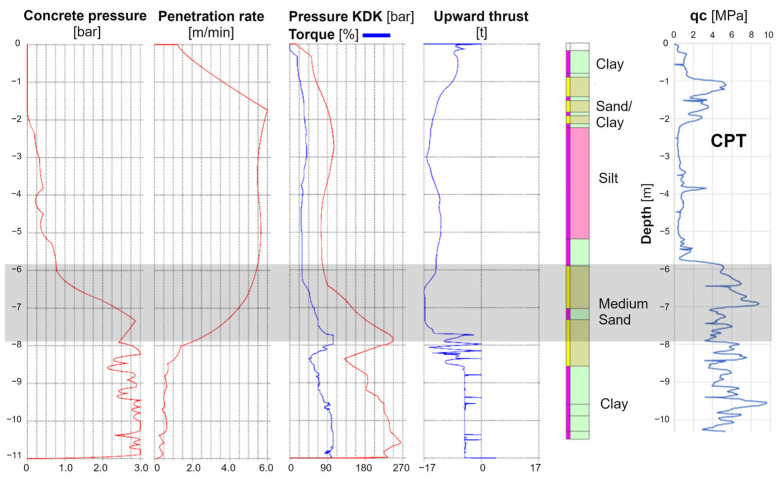
Comparison of the MWD pile drilling data set with the standard CPT results.

Some MWD data is obtained by automatically recording pressure parameters in the hydraulic systems of drilling machines. The combination of decreasing penetration rates and increasing torque readings might indicate strong soil layer, as shown in Figure 2. Obtaining reliable data on the soil conditions during drilling requires the rotation speed and penetration rate to be maintained at constant values. In piling practice, the operator accelerates the drilling in weak layers and slows down in strong ones. This results in changes in the readings in the KDK drilling head’s hydraulic system, and the accuracy of MWD data based on the pressure readings may be lower.

**Figure 3:**
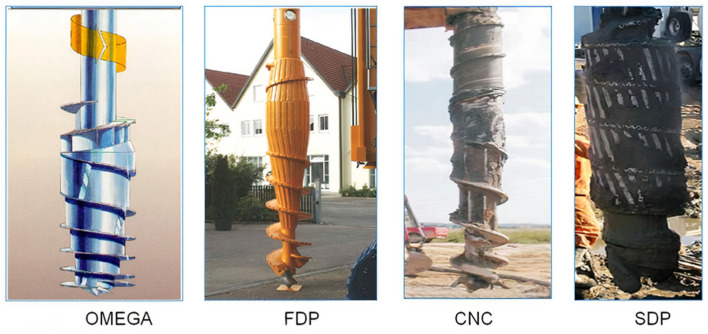
Examples of displacement augers.

In piling work, controlling the fresh concrete pressure is important to ensure the quality of pile base concreting. Unfortunately, MWD data is recorded by a sensor located at the top of a mast. This measurement is usually not accurate due to resistance of the concrete mix flow throughout the pump system. Poor pressure measurement accuracy and withdrawal of the auger too rapidly at the start of pile base concreting may reduce the load-bearing capacity of the pile (loosening the sandy soil under the pile toe), which is difficult to prove after completion of pile operations. This is particularly important when advanced displacement drilling bits are employed, as shown in Figure 3.

**Figure 4:**
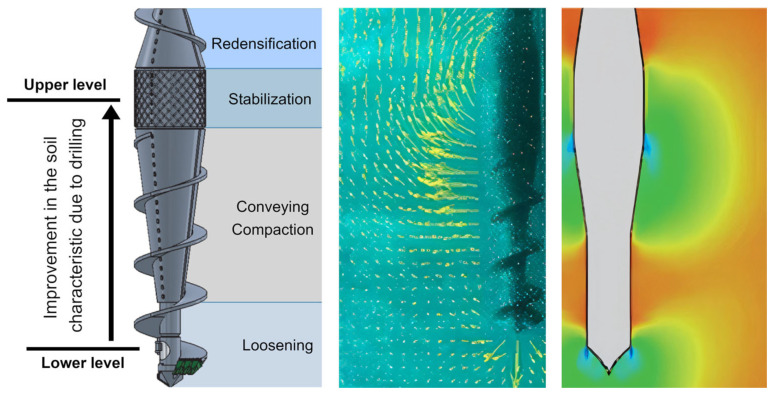
Pile–soil interaction based on numerical analyses of auger operation.

Drilled displacement pile technology is efficient in many aspects [13,24,25,26]. A significant improvement in the soil parameters during drilling with a displacement auger has been confirmed by numerous studies [27,28,29,30,31,32,33]. The auger’s impact on the ground during the drilling of a DDP is illustrated in Figure 4.

Despite the advantages of DDPs, a method for determining their load-bearing capacity has not yet been fully established [13,25,26,27,28,34,35,36,37,38]. Relatively little-understood issues in this field include the contribution of the pile side and base to the load transferred to the soil, as well as how to use CPT measurements to calculate the load-bearing capacity of the pile in the ground. This includes the challenge of accounting for the technological effects of drilling and concreting the pile in the ground. Studies by various authors indicate that design methods of the drilled displacement piles are currently not very accurate and only marginally consider the actual effect of the auger on the soil during drilling [13,17,25,28,31,32,33,36,37,38,39,40,41,42,43,44,45,46,47,48]. The authors’ experience indicates that the standard MWD systems installed in drilling rigs are used as a supplement to traditional field soil testing, providing a correlation between the parameters measured during drilling and well-known engineering properties of the soil.

## 3. Results

Contemporary challenges in advanced sensing technologies for geotechnical engineering are characterized by the need to transition from reactive methods based on experience to proactive methods based on measurement data and automated solutions. Geo-mechatronics is helpful in this context. This is a new field of knowledge within geotechnical engineering, combining mechanics, electronics, and robotics [49].

**Figure 5:**
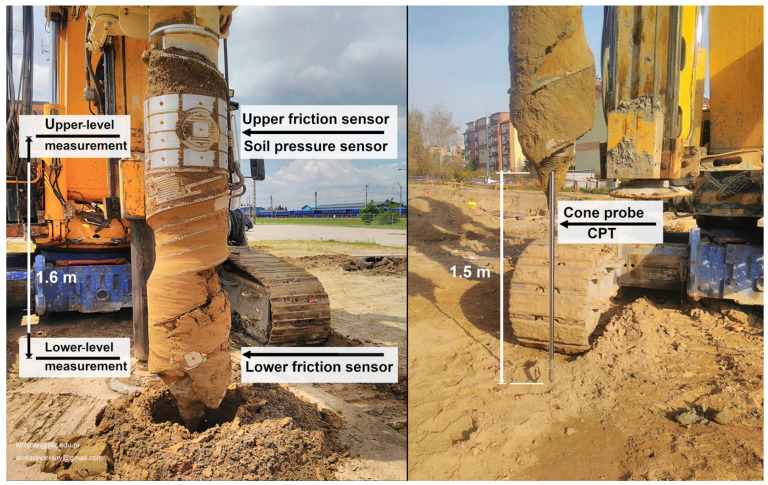
The new MWD-EGP drilling system with innovative sensors in the drill bit.

An example is the new MWD-EGP drilling system described in this article. Testing of this tool has been carried out in cooperation with Rzeszów University of Technology since 2017. Its practical applications were investigated using several specially designed, full-size prototypes of the EGP augers mounted on standard drilling rigs. The new system uses innovative sensors installed in the displacement drill bit. These sensors form an integrated control and measurement system and enable better real-time monitoring during pile drilling [50,51,52,53,54]. The main component of this system is a cutting-edge displacement auger EGP (Electro-Geo-Probe) drill, equipped with friction and soil pressure sensors and a CPT probe. A general view of the EGP drill system installed on a standard drilling rig is shown in Figure 5.

The key innovation in the new MWD-EGP drilling system lies in the placement of the sensors inside the drill bit (operating in the ground) rather than, as in other known standard MWD systems, in components of the drilling rig machine (operating at the surface of the construction site). An important advantage of the new drilling system is that the sensors are installed on the side wall of the auger at two levels with a vertical space of 1.6 m between them. This enables direct measurement of the soil resistance and assessment of the effects of soil compaction around the displacement pile during drilling. The MWD-EGP system is suitable for use with DDPs with diameters ranging from 500 mm to 750 mm and lengths up to 17 m.

**Figure 6:**
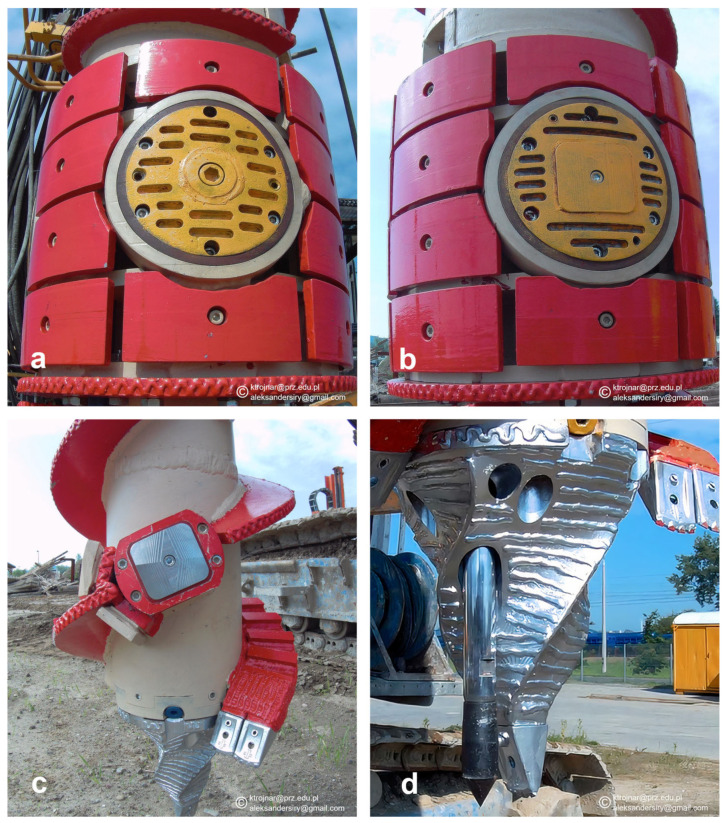
Sensors inside the EGP auger: (**a**). the soil pressure sensor at the upper level, (**b**). the friction sensor at the upper level, (**c**). the friction sensor at the lower level, (**d**). the CPT cone probe in the drill bit’s tip.

The most important measurement modules of the EGP system are as follows:

- **A module for measuring lateral soil pressure** on the auger, installed at the upper measurement level in a location where maximal soil compaction occurs during drilling, as shown in Figure 6a. The pressure sensor is at the same height as the upper friction sensor and is useful for measuring the horizontal soil pressure on the auger’s cylindrical surface in the compaction zone caused by displacement drilling. A measurement range of up to 1500 kPa allows for continuous monitoring of stress changes while drilling in most soil types. The measurement accuracy is 15 kPa.
- **A module for measuring soil frictional resistance** during pile drilling, installed at two levels on the auger, upper and lower, as shown in Figure 6b,c. The upper friction sensor is installed 160 cm above the lower one, in the zone of maximal lateral soil displacement during drilling. The tangential force values measured at this level are used to determine the friction resistance on the pile side in compacted soil zones. The measuring range is up to 500 kPa. The measurement has an accuracy of 5 kPa. Comparing the soil resistances recorded at both measurement levels allows for an assessment of the impact of displacement drilling in a specific cohesionless soil type. The lower friction sensor is installed on the side surface of the drill bit, approximately 20 cm above the blade, and has a measuring range of up to 500 kPa. The measurement has an accuracy of 5 kPa. The measured values reflect the characteristics of the native soil (without being affected by displacement drilling). Based on comparative EGPs field studies and standard CPTs, necessary correlations were established between the tangential force values of the friction sensors and the soil resistance on the pile side.
- **A module for measuring soil resistance below the drill bit tip** using a CPT probe that is pushed downward from inside the auger, as shown in Figure 6d. The CPT probe’s lower end is placed at an eccentric distance of 70 mm from the drill axis. It is equipped with a cone tip with a standardized diameter of 36.6 mm and an angle of 60 degrees. Using the periodically extended probe, the condition of the soil directly beneath the pile base can be examined. The standard extension speed is 2 cm/s (±2 mm/s). The maximum extension is 150 cm (±1 mm), and the measured resistance q.c does not exceed 50 MPa (max range up to 70 MPa). The measurement accuracy is 70 kPa.

The EGP system also includes other important components:

- A displacement drill bit with a specially designed spiral blade system for drilling in various soil types.
- A replaceable thermal head with an upper part designed to enclose and protect the electronic measurement systems installed inside.
- Air or water injection nozzles located at the bottom of the drill bit, useful for grouting beneath the pile base or overcoming resistance during drilling.
- A concrete pressure control module located at the end of the drill to monitor pile shaft formation in the soil.
- Control systems integrated with various modules in standard drilling rigs (hydraulic, electrical, and power transmission systems).
- Control modules connected to a central unit in the operator cabin, used to supervise the measurement subsystems and record, process, and analyze the data displayed on the monitor screen.
- A proprietary software package that enables real-time recording and analysis of drilling data.

**Figure 7:**
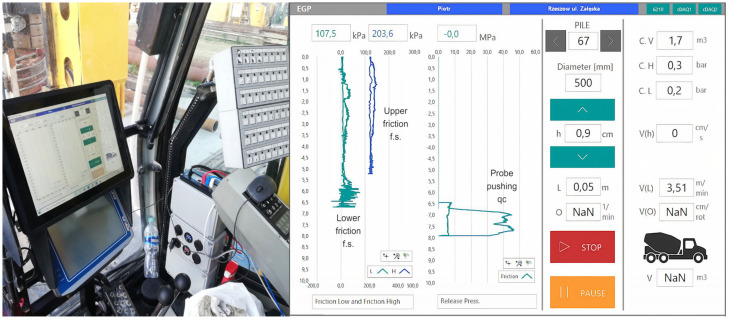
MWD-EGP system devices in the drill rig cabin and a preview on a monitor screen of the drilling results for a 6.5 m long and 500 mm diameter DDP.

During the pile drilling process, the operator uses an additional monitor in the cab to observe real-time graphs of the soil resistance at two drilling levels, as well as the CPT results obtained at a specific depth below the drill bit at the end of the drilling, as shown in Figure 7. All the EGP drilling data are recorded automatically and can be exported for further detailed analysis. The sampling frequency for each measurement track is higher than in standard drilling rigs and is 10 Hz. The operation of the drilling rig equipped with the EGP system is monitored via video cameras installed both outside and inside the operator’s cabin. This enables better coordination between the piling rig operator, the project engineer, and the designer, and even allows for ongoing remote observation of the on-site activity. An important advantage of the new system is its ability to remotely connect to the control panel in the operator’s cabin, allowing for real-time consultation on drilling-related issues. It is also possible to remotely control the CPT probe below the auger during the drilling process.

The advantages of the new MWD-EGP system over the existing NWD solutions are as follows:

- The measurements are carried out directly at the current drilling depth of the drill bit in the ground.
- The drill’s integration with a set of innovative sensors on the auger side surface and a CPT probe periodically extending from inside it allows for direct identification of the soil characteristics during drilling, with independent measurement of the soil resistance for the side wall and base of the drilled pile.
- The placement of the sensors at two levels on the drill bit allows for better control of the effect of pile installation in the ground in real time.
- The drilling resistance measurements on both the side wall and below the drill bit can be used for real-time verification of the subsoil layer location at the pile installation site.

The CPT sounding can be performed at the end of the EGP pile drilling phase to check that the drilling has been correctly (the operator’s decision) or even after the borehole has been partially filled with fresh concrete to confirm the soil resistance under the pile base (the engineer’s decision). The soil conditions below the pile base are crucial in determining the pile’s settlement under load. Existing pile calculation methods based on standard CPT probes recommend averaging the soil parameters above and below the pile base due to the uncertainty of designing, and difficulty of determining the effects of the pile installation in the ground (favorable or unfavorable) [16,17,33,34,35,36]. Improving the quality of MWD-EGP data obtained during pile drilling allows for the refinement of existing pile calculation methods. A separate article will propose a new method for calculating the load-bearing capacity of piles based on a direct CPT below the pile base during EGP drilling.

## 4. Discussion

### 4.1. Measurement of Pile Side Friction

**Figure 8:**
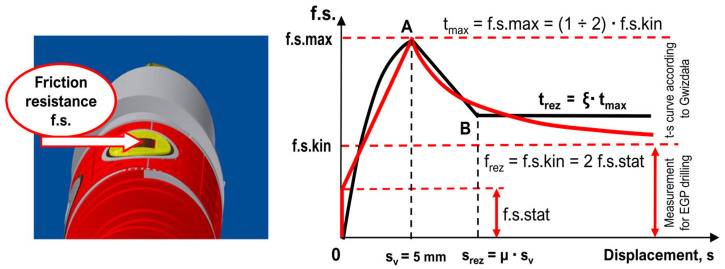
Characteristics of friction resistance in non-cohesive soil based on EGP measurements.

Comparative tests indicated that, in the initial phase of tangential force transfer from the EGP auger to the soil, static friction, f.s.stat, predominated. Stick–slip friction, f.s.max, appears first, and when displacements become large, the kinetic friction, f.s.kin, takes over. In some conditions, the kinetic friction values can be up to two times lower than the maximum static friction. Typically, this difference is smaller, and depending on the soil type, the kinetic friction ranges from 1.2 to 1.3. As the displacement increases, friction at the contact between the side wall and the soil may further decrease, even under the same normal stress. These findings are confirmed by other studies [55,56,57,58]. After reaching the limit of displacement, the kinetic friction stabilizes at a constant residual value. The graph of friction variation on the drill bit is similar to the t–s transform function described by Gwizdała [59,60]. The distribution of the frictional resistance on the side of the drill bit during drilling is shown in Figure 8.

**Figure 9:**
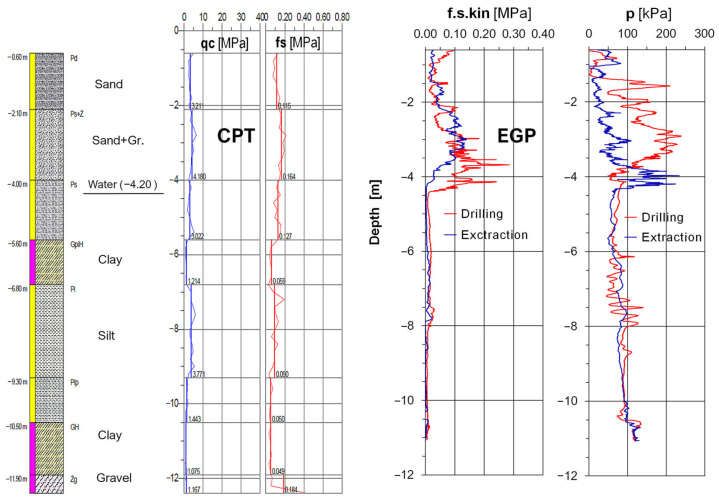
Comparison of the standard CPT data and the friction and soil pressure measured with the EGP sensors at the upper level.

Measurement of the horizontal soil pressure on the EGP auger when drilling in non-cohesive soils usually shows a difference in the values between downward (drilling) and upward drilling (extraction). A comparison of the soil pressure graphs in Figure 9 confirms the formation of a compaction zone around the EGP auger at a depth of 1–4 m. A comparison of the presented friction resistance values, f.s.kin, at the upper level of the EGP auger during drilling and extraction reveals a characteristic vertical shift in the maximum readings, confirming that displacement drilling causes not only lateral but also vertical soil movement. This observation suggests that non-cohesive soil’s behavior during displacement drilling may differ slightly from that observed during the conventional CPT.

**Figure 10:**
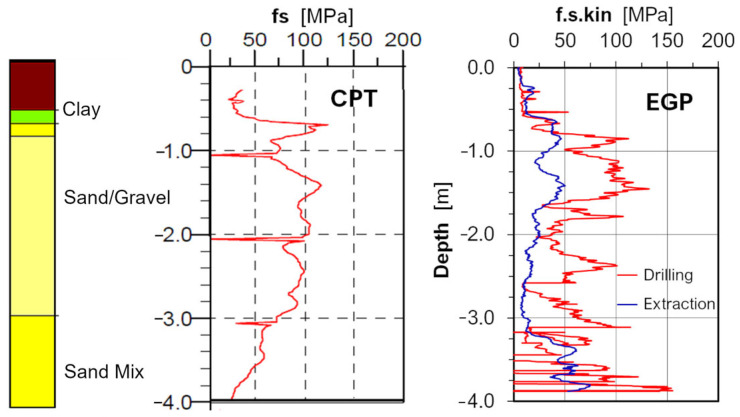
Comparison of the fs values obtained in the standard CPT and the friction measurements obtained using the EGP sensor at the lower level.

Figure 10 shows a comparison of the friction measured in sand by the lower sensor during drilling and extraction. The friction values measured when extracting the lower end of the drill bit are lower than those measured during downward drilling. This is due to the smaller diameter of the lower end of the drill bit. Additionally, this sensor moves up along the smooth inner surface of the drilled hole. Observations from the EGP drilling measurements confirm that the lower friction sensor only provides reliable values when drilling downwards.

### 4.2. Measurement of Pile Base Resistance

**Figure 11:**
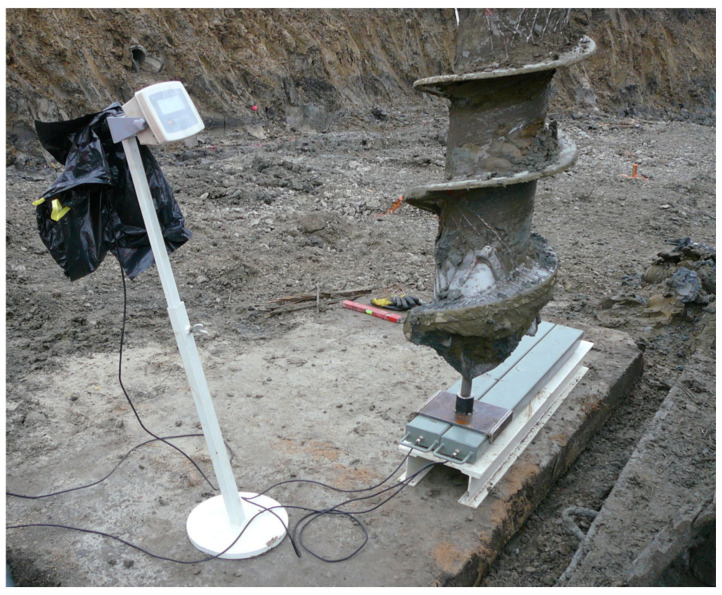
Calibration of the CPT probe inside the drill tool before starting the EGP pile series drilling.

A well-known key issue that has limited the integration of CPT probes in drills to date is soil degradation due to drilling. This has been considered a disadvantage of standard CPT sounding performed in boreholes, as drilling inherently degrades the in situ soil conditions. In the new MWD-EGP system, the CPT probe enables the automatic measurement of the “real” soil parameters, taking into account the influence of the drilling method and concreting of the pile. This is a significant advantage of the new EGP drilling solution compared to previous options, where standard CPT control soundings were performed near to the borehole, only after the pile had been concreted. The method of calibrating the CPT probe before the beginning of EGP drilling is shown in Figure 11.

Two practical examples illustrate the engineering benefits of using a CPT probe inserted into the native soil at a specified depth below an EGP pile’s base.

- **Example 1**—soil condition control at the final stage of pile drilling.

**Figure 12:**
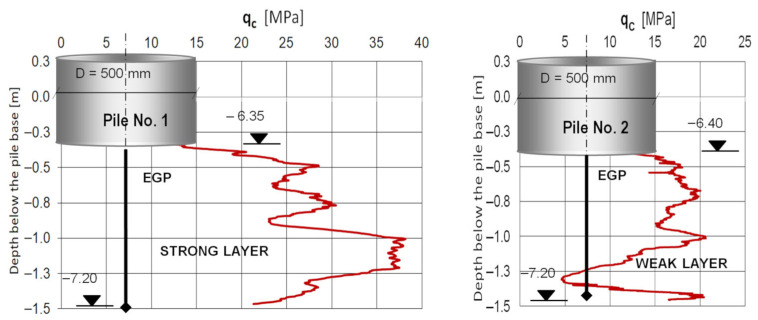
Comparison of measurements of the soil resistance under two adjacent EGP piles on the same construction site.

Figure 12 shows a control soil resistance test under two adjacent EGP piles on the same construction site, conducted just beyond the 0.4 m layer of fresh concrete embedded in the borehole of each pile. Each pile was 6.4 m long and 500 mm in diameter. The pile bases were designed on a strong layer of valley gravel mixed with sand. The purpose of this test was to verify whether the operator’s decision to terminate drilling at the same depth was equally correct for both piles. Comparison of the probing graphs for both piles shows that for pile No. 2, the soil approximately 1 m below the base was of lower quality than for pile No. 1. This indicates that the operator’s decision to stop drilling pile No. 2 at a depth of 6.4 m below ground level was incorrect. A better approach would likely have been to extend the pile by 1 m, repeat the CPT sounding, and complete the drilling in a soil layer with better geotechnical properties, in order to ensure comparable settlement under service load for both piles.

- **Example 2**—inspection of the ground conditions after the start of pile concreting.

**Figure 13:**
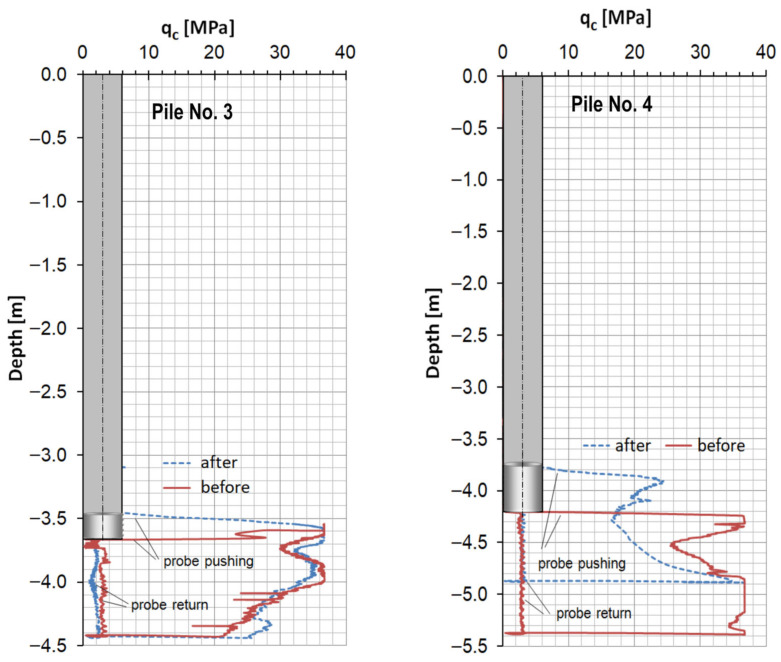
Measurement of the soil resistance under two EGP piles, before and after the start of concreting.

Figure 13 presents the CPT measurements beneath the two EGP piles, taken immediately before and after the start of fresh concrete deposited in the borehole. The purpose of this test was to check whether the concreting process could have a negative impact on the condition of the same cohesionless soil under the piles. A comparison of the graphs indicates that pile No. 3 was constructed correctly. In contrast, the concreting of pile No. 4 resulted in a deterioration in the soil quality beneath the pile base. As a result of the rapid extraction of the auger and the low pressure of the fresh concrete, the sandy soil under pile No. 4 was loosened at a depth of 4.2 m–4.7 m, which is visible as a difference in the EGP probing graphs obtained before and after the concreting of this pile began.

## 5. Summary

MWD techniques currently have great potential for use in geotechnical engineering. This paper presents the use of an innovative drilling method to improve quality control in the installation of drilled displacement piles in cohesionless soils. As a result, the drilling process can be actively and monitored in real time.

This is an example of the application of geo-mechatronics, a new engineering discipline that combines mechanics, electronics, and robotics within geotechnics. The improved use of data and automation of measurements using the new MWD-EGP drilling system represent a fundamental change in the approach to solving drilled displacement pile problems. It is a shift from traditional, often reactive and experience-based methods to proactive ones based on measurements and automated processes. The benefits include greater control of the drilling process, improved decision-making, and prevention of problems before they escalate.

Overall, the new approach reduces the drilling-related risks and enhances the safety of pile foundations. This innovative MWD-EGP drilling technique is an example of an implementation that is still being developed because it has great untapped potential.

In the near future, the integration of EGP measurement results with MWD data from standard drilling rigs using artificial intelligence may enable the development of new digital drilling rigs, which will be a powerful tool for comprehensive ground characterization in pile drilling under complex geological conditions.

## Data Availability

The data presented in this study are available on request from the corresponding author due to legal restriction.
